# Routine clinical variables do not support pre-endoscopic triage for gastric intestinal metaplasia in H. pylori-seropositive safety-net patients

**DOI:** 10.21203/rs.3.rs-11322056/v1

**Published:** 2026-10-27

**Authors:** Aaron Thrift, Nicholas Weaver, Neda Zarrin-Khameh, Ava Melmed, Tuesday Haynes, Jourdan Brown, Jennifer Mistica Zafiro Garcia-Villanueva, Lisa Danek, Hammad Mahmood, Thomas Porter, Noreen Pirzada, Jaime J. Rueda, Jay Orr, Fred Sutton, Jordan Sparkman, Maya Balakrishnan, Milena Suarez, Richa Shukla, Robert J. Sealock, Yanyan Chen, David Graham, Hashem El-Serag, Mimi Tan

**Affiliations:** Baylor College of Medicine

**Keywords:** Machine Learning, Gastric Intestinal Metaplasia, Helicobacter Pylori, Risk Prediction, Endoscopy

## Abstract

**Purpose:**

Gastric intestinal metaplasia (GIM) is a precursor to gastric adenocarcinoma and an actionable target for prevention. Pre-endoscopic risk scores may help prioritize endoscopy in heterogenous populations, but their additional value over *H. pylori* seropositivity alone remains uncertain. We evaluated whether routinely available clinical and demographic variables could identify GIM among *H. pylori*-seropositive adults in a safety-net setting.

**Methods:**

We analyzed 273 asymptomatic *H. pylori*-seropositive patients (GIM prevalence 28.9%) recruited from primary care clinics in a safety-net health system. Random forest (RF) and Least Absolute Shrinkage and Selection Operator (LASSO) models were developed using 8 pre-endoscopic predictors spanning demographics, clinical, lifestyle, and medical history factors. Performance was assessed in a held-out test set using area under the receiver operating characteristic curve (AUROC), classification metrics, and decision curve analysis (DCA).

**Results:**

Participants were predominantly Hispanic (92.3%), first-generation immigrants (90.5%), and low-income (72.9% with annual household income <$25,000). Age was the only candidate predictor associated with GIM (p = 0.033). RF and LASSO achieved held-out AUROCs of 0.558 (95% CI: 0.422–0.707) and 0.500 (95% CI: 0.371–0.643), respectively. Neither model provided clinically meaningful net benefit compared with endoscopic evaluation of all *H. pylori*-seropositive participants.

**Conclusion:**

In this high-risk safety-net population, routinely available pre-endoscopic variables did not meaningfully discriminate GIM after *H. pylori* seropositivity was established. Findings suggest risk-score-based triage may be insufficient to determine which seropositive patients should undergo endoscopy. Systematic endoscopic evaluation may be more appropriate in similar high-risk settings, while future prediction models may require biologic markers that better capture residual GIM risk.

## INTRODUCTION

Gastric cancer remains a major global health burden, with marked disparities in risk. In the United States, gastric cancer risk is concentrated among first-generation immigrants, Hispanic and other non-White populations, and socioeconomically disadvantaged groups, many of whom receive care in safety-net health systems where *Helicobacter pylori* infection is highly prevalent [1-4]. Gastric intestinal metaplasia (GIM) is a key precursor in the Correa cascade and represents a clinically actionable stage for endoscopic detection, surveillance, and gastric cancer prevention efforts [5-7].

Risk-based approaches to GIM screening have, therefore, focused on pre-endoscopic factors such as age, sex, race/ethnicity, birthplace, smoking, obesity, family history, and *H. pylori* infection status [8-14]. Existing models have shown moderate discrimination in heterogeneous populations, but much of their predictive signal derives from variables that distinguish individuals with high baseline population risk from those at lower risk, particularly *H. pylori* infection status which may underlie the predictive value of the other risk factors. These approaches are useful for identifying broad groups who may warrant testing or consideration for endoscopy, but they do not help clinicians identify individuals at high risk of GIM among those who are already *H. pylori* seropositive.

This question is especially important because endoscopic resources are limited and universal endoscopy for all *H. pylori* infected persons is unlikely to be feasible or cost-effective across many U.S. healthcare systems. However, if routinely available variables cannot distinguish seropositive patients with GIM from those without GIM, then risk score-based triaging may falsely reassure clinicians and lead to missed opportunities for gastric cancer prevention. Applying GIM risk stratification after a positive *H. pylori* test may provide a more stringent and clinically relevant strategy where pre-endoscopic data can support individualized endoscopy decisions after infection risk has already been established.

In this study, we aimed to develop and evaluate pre-endoscopy risk models for GIM among *H. pylori*-seropositive adults recruited from a high-risk safety-net primary care population. We hypothesized that routinely available clinical and demographic variables would have limited discrimination because the cohort was already enriched for the factors that have historically driven GIM prediction. Our goals were not only to build a model, but to determine whether model-based triage has sufficient clinical utility to justify prioritizing or deferring endoscopy in this setting.

## METHODS

### Design and Data Collection

As part of a gastric cancer screening study, we recruited participants aged 30–65 who were scheduled for routine outpatient visits at 8 participating primary care clinics within Harris Health, a safety-net health system in Harris County, Texas between February 12, 2021 and September 11, 2024. Recruitment, eligibility, and study flow have been described previously [15, 16]. Patients were excluded if they had prior *H. pylori* testing or treatment, prior gastrointestinal surgery or malignancy, or a contraindication to research endoscopy with gastric biopsy including bleeding disorders, pregnancy, anticoagulant use, or a BMI ≥ 40 kg/m^2^. Participants completed a study survey and a blood draw for *H. pylori* testing. The survey collected sociodemographic characteristics, immigration history and birthplace of the participant and their parents, lifetime history and current use of alcohol and smoking tobacco, history of reflux and dyspepsia symptoms, personal and family medical history, and medication use. Height, weight, and hip and waist circumference were measured. *H. pylori* serostatus was assessed using serum IgG ELISA; values > 0.89 were classified as positive, 0.80–0.89 as equivocal, and < 0.80 as negative. Seropositive participants were offered research esophagogastroduodenoscopy (EGD) with gastric biopsies obtained according to the Sydney protocol [17, 18]. Biopsies were obtained from five different anatomic locations: lesser curvature (A1) and greater curvature (A2) of the antrum, incisura angularis (A3), lesser curvature (C1) and greater curvature (C2) of the body. Hematoxylin and eosin (H&E) and Giemsa stain were used to assess *H. pylori* organisms on gastric biopsy samples. Active *H. pylori* infection was defined by histologic identification of organisms in at least one of five biopsy specimens; patients with no organisms identified at all five sites were deemed negative and excluded from further analysis. This study was approved by the Institutional Review Boards at Baylor College of Medicine and Harris Health. All participants provided written informed consent.

### Primary Outcome

The primary outcome was the histologic presence of GIM among *H. pylori*-seropositive participants. GIM was defined as intestinal metaplasia identified in at least 1 non-cardia gastric biopsy site and was considered absent only when intestinal metaplasia was absent from all biopsy sites. GIM extent was determined as All gastric biopsies were reviewed by a gastrointestinal pathologist blinded to the endoscopic findings and survey data.

### Model Features, Data Preprocessing, and Missing Data

Candidate predictors were selected *a priori* based on prior studies on GIM and clinical availability before endoscopy. The initial set included 33 variables across demographic characteristics, gastrointestinal symptoms, lifestyle factors, anthropometrics, and family and personal medical history (Online Resource 1). To preserve model stability given 79 GIM events and substantial demographic homogeneity, the final models included 8 predictors across four domains: demographic (sex, age group, generational status, and country or region of birth), anthropometric (body mass index [BMI] and waist-to-hip ratio), lifestyle (smoking), and family history of gastric cancer. Race/ethnicity, household income, education, gastrointestinal symptoms (presence, severity, and duration of stomach pain, bloating, heartburn, and regurgitation), age at migration to the U.S., parental birthplace, current smoking, current and lifetime alcohol and caffeine use, and additional family and personal medical history (family history of any cancer, family history of gastrointestinal disease, and personal history of gastrointestinal disease) were evaluated descriptively but not entered into the final models because of sparse cells, near-complete homogeneity, or limited events-per-variable. Age was dichotomized as 30–39 years versus 40–65 years. Personal history of gastric cancer was excluded prior to modelling because of near-zero variance (0 of 273 participants reported a positive history; 1 response was categorized as unknown). Country of birth was collapsed into clinically interpretable regions to address low counts: Central America (Honduras, El Salvador, Guatemala, Nicaragua), South America (Colombia, Ecuador, Venezuela), and Other (all remaining countries, each with n ≤ 3). The full predictor list is provided in Table 1.

Missingness was low for most predictors (0%-2.6%); waist-to-hip ratio had the highest missingness (11.7%, n = 32). Missing categorical values were imputed with the mode before model training. Univariate associations between candidate predictors and GIM status were assessed using chi-square tests, with Fisher's exact test using Monte Carlo simulation when expected cell counts were < 5. All analyses were performed using R version 4.5.1.

### Model Development and Evaluation

We fit two complementary supervised learning models: LASSO penalized logistic regression and random forest (RF) classification [19, 20]. LASSO was selected to provide shrinkage, variable selection, and interpretable coefficients in a modest-sized cohort, whereas RF was selected to evaluate whether nonlinear relationships or interactions could capture risk patterns missed by regression-based modelling.

The dataset was split into a training set (70%) and held-out test set (30%) using stratified random sampling to preserve GIM prevalence. Model development and hyperparameter tuning were performed only within the training set, and the held-out test set was reserved for final performance evaluation.

The RF model was trained with nested 5-fold cross-validation for hyperparameter selection and internal performance estimation. Inverse class weights were applied to address the lower prevalence of GIM relative to non-GIM. The final RF model was then refit on the full training set using the selected hyperparameters and evaluated in the held-out test set.

For the LASSO model, categorical predictors were dummy coded before fitting. The regularization parameter lambda was selected using 10-fold cross-validation, with the one-standard-error lambda used as the primary model to reduce overfitting. Retained predictors were summarized using model coefficients on the log-odds scale.

Model performance in the held-out test set was evaluated using the area under the receiver operating characteristics (AUROC) curve, sensitivity, specificity, positive predictive value (PPV), and negative predictive value (NPV), with the classification threshold selected by Youden's J index. Bootstrap confidence intervals (CIs) were calculated for all performance metrics. Clinical utility was evaluated using decision curve analysis (DCA), which estimates the net benefit of a prediction strategy across clinically relevant threshold probabilities and compares model-based triage with default strategies of performing endoscopy on all patients or none [21].

## RESULTS

### Sample Characteristics

A total of 273 participants seropositive for *H. pylori* were included in the analysis, including 79 (28.9%) who had biopsy-confirmed GIM (Table 1). The cohort was predominantly Hispanic (92.3%), female (75.1%), and first-generation immigrants (90.5%) with the majority being born in Mexico (47.3%) or Central America (39.2%). Most participants had less than a high school education (66.7%) and an annual household income below $25,000 (72.9%). Among GIM positive patients, 22 had extensive GIM, while 57 had antrum-only GIM with no cases having OLGIM stage III or IV. Age was the only selected predictor variable associated with GIM (p = 0.033); GIM prevalence was 17.9% among participants aged 30–39 years and 32.5% among those aged 40–65 years. No other selected predictor was significantly associated with GIM (all p > 0.10).

### Model Performance

Both LASSO and RF models demonstrated poor discrimination, indicating that routinely available pre-endoscopic variables did not identify a clinically useful distinction between participants with and without GIM (Table 2).

For the RF model, mean AUROC across nested 5-fold cross-validation was 0.518 (95% CI: 0.324–0.712). In the held-out test set, the RF model achieved an AUROC of 0.558 (95% CI: 0.422–0.707), indicating poor discrimination. Sensitivity was low (0.288; 95% CI 0.175–0.397) and specificity was modest (0.542; 95% CI 0.321–0.739), suggesting that the model did not reliably identify patients with GIM.

The LASSO model similarly failed to discriminate GIM status. Cross-validation AUROC was 0.532 (95% CI 0.444–0.620) and held-out test set AUROC was 0.500 (95% CI: 0.371–0.643). LASSO retained 5 of 10 dummy-coded predictors: age 40–65 years, third-generation immigrant status, high waist-to-hip ratio, female sex, and BMI 25–<30 kg/m^2^. Although the directions of several coefficients were clinically plausible (Table 3), coefficient magnitudes were small and did not result in meaningful improvements in discrimination.

DCA demonstrated no clinically meaningful net benefit of risk-based triage over universal endoscopic evaluation (the Treat All reference strategy) across threshold probabilities of 5%–50% (Fig. 1). At a threshold of 10%, the RF model, LASSO model, and Treat All strategy produced equivalent net benefit. Between thresholds of 20–27%, the LASSO model demonstrated marginally higher net benefit than universal endoscopy, although this difference may reflect sampling variability in the held-out test set. At the observed GIM prevalence threshold of 29%, both models yielded negative net benefit, falling below the Treat All reference. Above 30%, all strategies produced negative or near-zero net benefit. The Treat All strategy maintained positive net benefit at referral thresholds below the population GIM prevalence of 28.9%. Across both modelling approaches and DCA, results were consistent as neither model identified a clinically useful decision boundary for pre-endoscopic GIM prediction in this population. The consistency across modelling approaches suggests that the poor discrimination reflects characteristics of the study population rather than a single modelling strategy.

## DISCUSSION

In this study of 273 *H. pylori*-seropositive adults recruited from a safety-net primary care population, routinely available clinical and demographic variables did not meaningfully predict biopsy-confirmed GIM. Both RF and LASSO models produced AUROCs near chance in held-out testing, and DCA showed no clinically useful net benefit for model-based triage compared with endoscopic evaluation of all *H. pylori*-seropositive participants. These findings suggest that, in a population with widespread *H. pylori* infection and demographic risk, the routine pre-endoscopic variables do not appear sufficient to determine which patients can safely defer endoscopy.

These findings clarify the limitations of prior GIM risk models rather than contradict them. Previous models derived in heterogeneous U.S. populations have demonstrated that pre-endoscopic demographic and clinical variables can stratify GIM risk. Tan et al. developed a risk prediction model pooling data from a Veterans Affairs hospital and several Catholic Health Initiative St. Luke’s Hospitals [13, 14]. This model incorporated sex, age, race/ethnicity, smoking, and *H. pylori* infection and achieved an AUROC of 0.74 in the pooled validation cohort for predicting GIM. In another study of a multiethnic safety-net cohort, older age, non-White race/ethnicity, birthplace, and smoking were independently associated with GIM, whereas *H. pylori* infection was not independently associated with GIM [12]. This was attributed partly to the high prevalence of *H. pylori* infection in both cases and controls, limiting its discriminatory value. Haghighat et al. similarly identified country of birth, age, race/ethnicity, active *H. pylori* infection, and smoking as predictors [22]. That study also demonstrated substantial variation in GIM risk within Latin America. In contrast, our cohort was restricted to *H. pylori*-seropositive patients and consisted predominantly of first-generation Hispanic immigrants from Mexico and Central America. Under these conditions, many variables that are predictive across heterogeneous populations become less informative because most participants already occupy a high-risk stratum.

This distinction is clinically important. For a clinician caring for a safety-net population, the relevant decision is not whether Hispanic or immigrant patients are at increased risk overall, but whether a specific *H. pylori*-seropositive patient should undergo endoscopy to detect GIM. Conversely, which patients can defer endoscopy. Our results suggest that survey-based demographics, symptoms, anthropometrics, smoking history, and family history do not provide sufficient incremental information to drive those decisions. Thus, the poor performance of the models is itself clinically informative, indicating that routine pre-endoscopy data have limited value as a triage tool once *H. pylori* seropositivity is identified as high-risk subgroup.

Restricted residual heterogeneity provides a plausible explanation. *H. pylori* infection is a central driver of gastric carcinogenesis, and prior demographic predictors may partly operate as markers of infection acquisition, early-life exposure, environmental context, or other population-level risks. When infection status and demographic risk are relatively homogenous, remaining variation in GIM risk may depend on factors not captured by routine clinical data, including duration of infection, bacterial virulence, host inflammatory and genetic susceptibility, gastric microbiome composition, and serologic markers of atrophy [23, 24]. Incorporating such biologic factors may therefore be necessary to improve risk discrimination among *H. pylori* infected individuals. The high prevalence of GIM in this cohort further supports the clinical relevance of these findings. Nearly one in three *H. pylori*-seropositive participants undergoing research endoscopy had GIM, despite recruitment from primary care rather than symptom-driven gastroenterology referral population. Gastrointestinal symptoms did not distinguish GIM cases from non-cases, further limiting their value for triage. If confirmed prospectively, these findings support consideration of a lower threshold for endoscopic evaluation among *H. pylori*-seropositive patients in similar high-risk safety-net populations, while recognizing that endoscopic capacity, cost-effectiveness, and patient preferences must be considered.

Our study has several strengths. It addresses a practical and underexplored clinical question regarding whether conventional pre-endoscopic risk stratification adds value within a population already at an elevated risk for GIM. The cohort’s demographic concentration, while limiting generalizability, allowed a focused test of residual risk prediction within a population highly relevant to gastric cancer prevention. We used IgG serology to initially screen for *H. pylori* infection then used biopsy histopathology to confirm presence of active infection. We used two complementary modelling approaches, cross-validation, held-out testing, bootstrap CIs, and DCA to evaluate both discrimination and clinical utility. The consistently poor performance across these approaches suggests that the findings are not attributable to a single modelling strategy.

Several limitations should be considered. The sample included 273 participants and 79 GIM cases, limiting power and precision. Larger studies identify smaller predictive signals. As a cross-sectional study of patients presenting for primary care visits, the cohort may not represent all *H. pylori*-seropositive individuals particularly those less engaged with healthcare. Country of birth granularity was reduced to regional groupings due to small cell sizes, potentially obscuring within-region heterogeneity in GIM risk. The Lauren classification defines two histological subtypes of gastric adenocarcinoma: intestinal-type and diffuse-type [25]. This study examined participants with intestinal-type precursor lesions; thus, findings cannot be applied to patients with diffuse-type adenocarcinoma. Factors such as CagA status, pepsinogen I and II levels, salt intake, history of gastric ulcer, and ascorbic acid intake were not collected. Additionally, the held-out test set comprised only 83 participants with 24 GIM-positive cases, producing wide CIs that limit the precision of individual performance estimates; however, consistent findings across nested cross-validation, held-out testing, and DCA supports the overall conclusion. Finally, the cross-sectional design precluded assessment of GIM progression, regression after *H. pylori* treatment, or incident gastric cancer.

In conclusion, among *H. pylori*-seropositive adults in a high-risk safety-net primary care setting, routinely available clinical and demographic variables did not support meaningful pre-endoscopic risk stratification for GIM. Once *H. pylori* seropositivity identifies a demographically high-risk population, conventional clinical variables may provide insufficient information to determine who can safely defer endoscopy. These findings support consideration of systematic or lower-threshold endoscopic evaluation in similar populations, pending prospective validation and cost-effectiveness analyses. Future prediction models should move beyond routine survey and clinical variables to incorporate biologic markers that may better capture residual GIM risk among *H. pylori* infected individuals.

## Supplementary Material

This is a list of supplementary files associated with this preprint. Click to download.

• TOPSM1.pdf

## Figures and Tables

**Figure 1:**
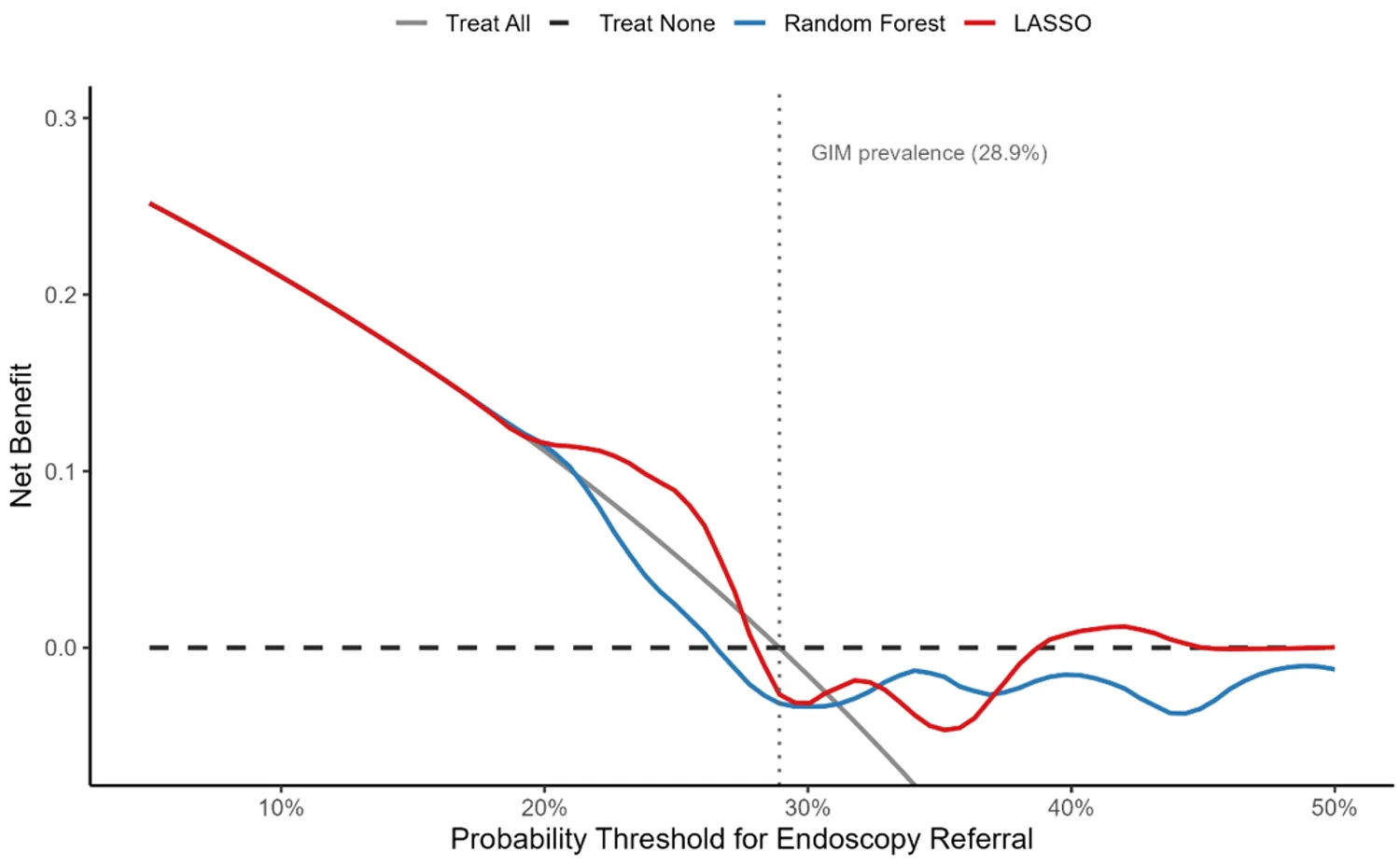
Decision curve analysis for prediction of gastric intestinal metaplasia in the held-out test set Net benefit is plotted across probability thresholds for endoscopy referral, ranging from 5% to 50%. The solid grey line ("Treat All") represents referring all H. pylori-seropositive patients for endoscopy regardless of predicted risk; the dashed black line ("Treat None") represents referring no patients. The blue and red lines represent the random forest (RF) and least absolute shrinkage and selection operator (LASSO) models, respectively. The vertical dotted line marks the observed GIM prevalence in the cohort (28.9%). Neither model exceeded the net benefit of the Treat All strategy at any clinically relevant threshold; the Treat All strategy maintained positive net benefit at referral thresholds below the observed GIM prevalence. n=83 (held-out test set). GIM, gastric intestinal metaplasia.

**Table 1:** Characteristics of *H. Pylori*-Seropositive Study Participants Stratified by GIM Status

Variable	Overall (n = 273)	No GIM (n = 194)	GIM (n = 79)	P-value
**Sex**				**0.384**
Male	68 (24.9%)	45 (23.2%)	23 (29.1%)	
Female	205 (75.1%)	149 (76.8%)	56 (70.9%)	
**Age**				**0.033**
30–39	67 (24.5%)	55 (28.4%)	12 (15.2%)	
40–65	206 (75.4%)	139 (71.6%)	67 (84.8%)	
**Generational Status**				**0.17** ^a^
First Generation	247 (90.5%)	176 (90.7%)	71 (89.9%)	
Second Generation	6 (2.2%)	6 (3.1%)	0 (0%)	
Third Generation	20 (7.3%)	12 (6.2%)	8 (10.1%)	
**Body Mass Index (kg/m^2)**				**0.745**
18.5 ≤ 25	30 (11%)	20 (10.3%)	10 (12.7%)	
25 ≤ 30	98 (35.9%)	68 (35.1%)	30 (38%)	
30 ≤ 35	80 (29.3%)	60 (30.9%)	20 (25.3%)	
35+	58 (21.2%)	43 (22.2%)	15 (19%)	
Missing	7 (2.6%)	3 (1.5%)	4 (5.1%)	
**Waist-to-Hip Ratio**				**0.555**
Normal	45 (16.5%)	30 (15.5%)	15 (19%)	
High	196 (71.8%)	142 (73.2%)	54 (68.4%)	
Missing	32 (11.7%)	22 (11.3%)	10 (12.7%)	
**Country of Birth**				**0.755** ^a^
Mexico	129 (47.3%)	90 (46.4%)	39 (49.4%)	
Central America	107 (39.2%)	76 (39.2%)	31 (39.2%)	
**Sex**				**0.384**
South America	5 (1.8%)	5 (2.6%)	0 (0%)	
USA	26 (9.5%)	18 (9.3%)	8 (10.1%)	
Other	6 (2.2%)	5 (2.6%)	1 (1.3%)	
**Smoking History**				**0.203**
Never	220 (80.6%)	162 (83.5%)	58 (73.4%)	
Ever	46 (16.8%)	29 (14.9%)	17 (21.5%)	
Missing	7 (2.6%)	3 (1.5%)	4 (5.1%)	
**Family History of Gastric Cancer**				**0.188** ^a^
No	264 (96.7%)	187 (96.4%)	77 (97.5%)	
Yes	6 (2.2%)	6 (3.1%)	0 (0%)	
Missing	3 (1.1%)	1 (0.5%)	2 (2.5%)	

GIM gastric intestinal metaplasia, BMI body mass index

P-values from chi-square test unless otherwise noted.

a Fisher's exact test with Monte Carlo simulation (10,000 replicates) performed due to sparse expected cell counts (< 5).

**Table 2:** Model Performance: Random Forest vs. LASSO for GIM Prediction

Metric	Random Forest	LASSO
Internal Validation: Nested CV AUROC (95% CI)	0.518 (95% CI: 0.324–0.712)	0.532 (95% CI: 0.444–0.62)
Test set AUROC (95% CI)	0.558 (95% CI: 0.422–0.707)	0.5 (95% CI: 0.371–0.643)
Test set Sensitivity (95% CI)	0.288 (95% CI: 0.175–0.397)	0.576 (95% CI: 0.446–0.695)
Test set Specificity (95% CI)	0.542 (95% CI: 0.321–0.739)	0.292 (95% CI: 0.111-0.5)
Test set PPV (95% CI)	0.607 (95% CI: 0.419–0.774)	0.667 (95% CI: 0.527–0.796)
Test set NPV (95% CI)	0.236 (95% CI: 0.125–0.354)	0.219 (95% CI: 0.08–0.36)
N predictors entered	8	5 of 10 dummy columns

GIM gastric intestinal metaplasia, CI confidence interval, PPV positive predictive value, NPV negative predictive value, CV cross validation, AUROC area under the receiver operator characteristic

Internal validation via nested 5-fold cross-validation (Random Forest) and 10-fold cross-validation (LASSO). Test set metrics derived from a stratified 30% held-out sample (n = 83) with 1,000-resample bootstrap 95% confidence intervals. Classification threshold selected by Youden's J index.

**Table 3:** LASSO Coefficients for GIM Prediction

Predictor	Coefficient	Direction
Waist-to-Hip Ratio (High vs. Normal)	−0.384	Lower GIM risk
Age (40–65 vs. 30–39 years)	0.372	Higher GIM risk
Generational Status (Third vs. First Generation)	0.260	Higher GIM risk
Sex (Female vs. Male)	−0.142	Lower GIM risk
BMI (25 ≤ 30 vs. 18.5 ≤ 25 kg/m^2^)	−0.133	Lower GIM risk

GIM gastric intestinal metaplasia

Coefficients on the log-odds scale; reference categories are the comparator level shown in parentheses.

Positive coefficients indicate association with higher predicted GIM probability.

## Data Availability

Data may be available to other researchers with IRB approved protocols by contacting the corresponding author (APT).
